# A novel nomogram for decision-making assistance on exemption of axillary lymph node dissection in T1–2 breast cancer with only one sentinel lymph node metastasis

**DOI:** 10.3389/fonc.2022.924298

**Published:** 2022-09-12

**Authors:** Lei Liu, Yaoxin Lin, Guozheng Li, Lei Zhang, Xin Zhang, Jiale Wu, Xinheng Wang, Yumei Yang, Shouping Xu

**Affiliations:** ^1^ Department of Breast Surgery, Harbin Medical University Cancer Hospital, Harbin, China; ^2^ Chinese Academy of Sciences (CAS) Center for Excellence in Nanoscience, Chinese Academy of Sciences (CAS) Key Laboratory for Biomedical Effects of Nanomaterials and Nanosafety, National Center for Nanoscience and Technology, Beijing, China; ^3^ Department of The First Operating Room, The Second Affiliated Hospital of Harbin Medical University, Harbin, China

**Keywords:** breast cancer, axillary lymph node dissection, exemption, nomogram, non-sentinel lymph node

## Abstract

**Background:**

T1–2 breast cancer patients with only one sentinel lymph node (SLN) metastasis have an extremely low non-SLN (NSLN) metastatic rate and are favorable for axillary lymph node dissection (ALND) exemption. This study aimed to construct a nomogram-based preoperative prediction model of NSLN metastasis for such patients, thereby assisting in preoperatively selecting proper surgical procedures.

**Methods:**

A total of 729 T1–2 breast cancer patients with only one SLN metastasis undergoing sentinel lymph node biopsy and ALND were retrospectively selected from Harbin Medical University Cancer Hospital between January 2013 and December 2020, followed by random assignment into training (n=467) and validation cohorts (n=262). A nomogram-based prediction model for NSLN metastasis risk was constructed by incorporating the independent predictors of NSLN metastasis identified from multivariate logistic regression analysis in the training cohort. The performance of the nomogram was evaluated by the calibration curve and the receiver operating characteristic (ROC) curve. Finally, decision curve analysis (DCA) was used to determine the clinical utility of the nomogram.

**Results:**

Overall, 160 (21.9%) patients had NSLN metastases. Multivariate analysis in the training cohort revealed that the number of negative SLNs (OR: 0.98), location of primary tumor (OR: 2.34), tumor size (OR: 3.15), and lymph-vascular invasion (OR: 1.61) were independent predictors of NSLN metastasis. The incorporation of four independent predictors into a nomogram-based preoperative estimation of NSLN metastasis demonstrated a satisfactory discriminative capacity, with a C-index and area under the ROC curve of 0.740 and 0.689 in the training and validation cohorts, respectively. The calibration curve showed good agreement between actual and predicted NSLN metastasis risks. Finally, DCA revealed the clinical utility of the nomogram.

**Conclusion:**

The nomogram showed a satisfactory discriminative capacity of NSLN metastasis risk in T1–2 breast cancer patients with only one SLN metastasis, and it could be used to preoperatively estimate NSLN metastasis risk, thereby facilitating in precise clinical decision-making on the selective exemption of ALND in such patients.

## Introduction

Breast cancer, the most common cancer in women, has developed rapidly around the world in recent years, posing a serious threat to human health. According to the global cancer statistics published by the International Agency for Research on Cancer in 2020, the incidence of breast cancer is as high as 2.26 million, surpassing lung cancer to become the world’s leading cancer (1). At the same time, 684,996 deaths also made breast cancer the leading cause of cancer deaths in women worldwide in 2020 (1).

As we know, axillary lymph node status is an essential and critical factor in the assessment of breast cancer staging, prognosis, and subsequent treatment. Sentinel lymph node biopsy (SLNB) can accurately evaluate the axillary lymph node status of breast cancer patients with negative clinical axillary lymph nodes (2) and has been widely implemented in clinical applications. In patients with sentinel lymph node (SLN) metastases, axillary lymph node dissection (ALND) is usually unavoidable, despite several postoperative complications, such as the limitation of the activity of the articulatio humeri, upper limb edema, and neuropathic pain (3). Unfortunately, previous studies have shown that up to 60% of breast cancer patients with SLN metastases have no further non-SLN (NSLN) metastases in ALND (4–6), and this rate was even higher in T1–2 breast cancer patients (7–14), which means that ALND is completely unnecessary in more than half of these patients. In addition, the value of ALND in the decision-making of adjunctive treatment is increasingly being questioned. Those have led to a heated debate over whether ALND needs to be routinely performed in breast cancer patients with SLN metastases, and the voice of ALND-selective exemption in such patients is getting more and more affirmative responses.

The Memorial Sloan–Kettering Cancer Centre (MSKCC) nomogram developed by Kimberly Van Zee and colleagues in 2003 was the first model for predicting the risk of NSLN metastases in breast cancer patients with SLN metastases (15). Although the area under the curve (AUC) of the nomogram was 0.76 in the Kimberly Van Zee study, indicating satisfactory accuracy and discrimination capacity, subsequent validation results in other cohorts gave suboptimal results (16–18). Since then, some other nomograms have been proposed, such as nomograms from Tenon (19), Cambridge (20), Stanford (21), and Helsinki University (22). In addition, several clinical trials represented by ACOSOG Z0011, IBCSG 23-01, and AMAROS have been carried out and proved that in breast cancer patients with limited SLN involvement, further ALND treatment had no significant effect on Disease-free survival (DFS) and Overall survival (OS) (23–25). Based on the results of these clinical trials, the NCCN guidelines also recommended not performing ALND in breast cancer patients with one to two SLN metastases who will undergo breast-conserving surgery and subsequent radiotherapy (26). However, the proportion of breast-conserving surgery in developing countries represented by China is generally low, less than 30% (27, 28), compared with 50%–80% in developed countries (29). As a result, this recommendation faces significant limitations in clinical application, especially in developing countries. ALND is still recommended for most breast cancer patients with SLN metastasis, even if some of them will not benefit at all.

Moreover, early breast cancer patients with low axillary tumor burden, especially for T1–2 breast patients with only one SLN metastasis, theoretically have a lower axillary tumor burden and risk of NSLN metastasis. Previous studies have shown that the NSLN metastatic rate in breast cancer patients with only one SLN metastasis is approximately 25%–35% (30, 31), while the NSLN metastatic rate in T1–2 breast cancer patients with SLN metastases is approximately 30%–40% (32, 33). Therefore, as the most potential competitors for selective exemption of ALND, the predictors and prediction models of NSLN metastasis in T1–2 breast cancer patients with only one SLN metastasis need further research. However, in previous studies, such targeted studies remain scarce. In this study, we analyzed the clinicopathological information of T1–2 breast cancer patients with only one SLN metastasis to explore the NSLN metastasis–related independent predictors in such patients and aimed to construct and validate a preoperative prediction model for the risk of NSLN metastasis.

## Materials and methods

### Patients and data collection

This study retrospectively analyzed T1–2 breast cancer patients who underwent SLNB and ALND at the Harbin Medical University Cancer Hospital between January 2013 and December 2020. In addition, all of them were confirmed to have only one SLN metastasis. Major exclusions were a tumor size more than 5 cm, treated with neoadjuvant chemotherapy, complicating with other cancers, or lacking clinicopathological information. Finally, a total of 729 patients were included in this study. Additionally, those patients were randomly assigned to the training cohort (n=467) and the validation cohort (n=262).

The medical records of eligible patients were reviewed, and clinicopathological information was collected, including age, carcinoembryonic antigen (CEA), carbohydrate antigen 153 (CA153), distance from nipple, surgical approach, location of the primary tumor, primary histologic type, clinical tumor size, molecular subtypes [luminal A, luminal B, triple-negative, or human epidermal receptor-2 (HER-2) overexpression; these were according to estrogen receptor (ER), progesterone receptor (PR), and HER-2 statuses, and the Ki-67 index], lymph-vascular invasion (LVI), and the number of negative SLNs (negSLN).

### Sentinel lymph node biopsy and axillary lymph node dissection

All patients included in the present study underwent SLNB and ALND. SLNB is performed using one of the following tracer techniques: methylene blue, combined methylene blue–radioactive tracer, or combined methylene blue–fluorescence. All these tracer techniques have a high detection rate and low false-negative rate (34). SLNs were examined by intraoperative frozen sections, and only one SLN metastasis for each patient was confirmed. Immunohistochemistry is not routinely used in the diagnosis of SLN unless it is required. After the pathological confirmation of SLN metastasis, all patients underwent ALND. Dissection included all level I–III axillary nodes. For all nodes acquired by ALND, a routine paraffin pathological examination was performed. All nodes were H&E-stained and reviewed by two experienced pathologists. According to the American Joint Committee on Cancer Staging Manual (7th edition), positive nodes were defined as having a metastasis size greater than 0.2 mm and/or more than 200 malignant cells.

### Statistical analysis

Continuous variables were shown as means ± standard deviation, which were compared by Student’s t-test, while categorical variables were shown as frequencies and proportions and compared with the chi-square and Fisher’s exact tests. The possible predictors associated with NSLN metastasis were analyzed by univariate analysis in the training cohort. Then, multivariable logistic regression analysis was conducted based on the significant variables (p<0.05) in univariate analysis to identify the independent predictors of NSLN metastasis. The odds ratio (OR) and corresponding 95% confidence interval (CI) were calculated and presented. The above statistical analyses were performed using IBM SPSS version 24.0 (SPSS Inc., Chicago, IL, USA).

The nomogram was established based on the independent predictors of NSLN metastasis in the multivariate analysis. The discrimination and accuracy of the nomogram-based prediction of the NSLN metastasis risk were evaluated by the concordance index (C-index) and calibration curves in both training and validation cohorts. Furthermore, receiver operating characteristic (ROC) curves were plotted, followed by the calculation of the AUC of ROC curves, aiming to investigate the overall performance of this nomogram. The C-index and AUC over 0.7 indicate that the nomogram provides a reasonable estimation. Decision curve analysis (DCA) was established to assess the clinical validity of the nomogram. The above statistical analyses were performed by R4.1.0 software with rms, survival, readr, foreign, and rmda package.

## Results

### Clinicopathological characteristics

**Figure 1:**
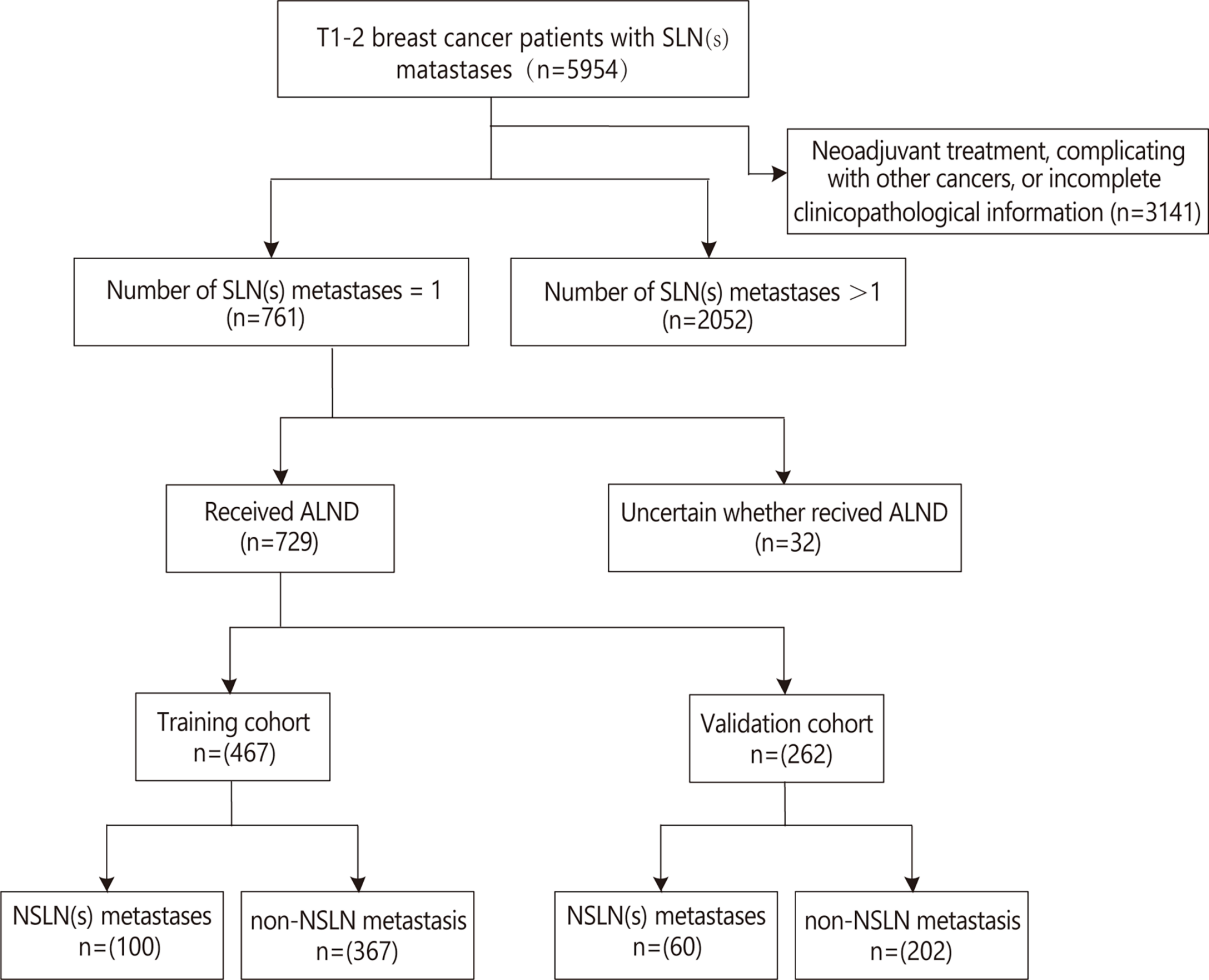
Flow diagram for the study. ALND, axillary lymph node dissection; NSLN, non-sentinel lymph node; SLN, sentinel lymph node.

There were a total of 5,954 T1–2 breast cancer patients with SLN metastases during the review period, and 3,141 cases of them were excluded because of the exclusion criteria. Of the remaining 2,813 patients, 761 had only one SLN metastasis. Of these 761 patients, 32 cases were uncertain whether they received ALND and were excluded. Finally, 729 patients with T1–2 breast cancer who had only one SLN metastasis and underwent ALND were included and were randomly divided into the training cohort and the validation cohort set in a 2:1 ratio (**Figure 1**). Their mean age was 53.84 ± 10.03 years in the training cohort and 54.95 ± 9.15 years in the validation cohort. Their NSLN metastatic rate was 21.4% (100/467) in the training cohort and 22.9% (60/262) in the validation cohort. The baseline clinicopathological characteristics of the patients in both cohorts are summarized in **Table 1**. Generally, there was no significant difference in the clinicopathological information between the training cohort and the validation cohort.

**Table 1:** Clinicopathological characteristics of patients.

Clinicopathological characteristics		Training cohort (n=467)	Validation cohort (n=262)	Whole cohort	*χ^2^/t*	p-value
**Age (years)**		53.84 ± 10.03	54.95 ± 9.15	54.24 ± 9.74	-1.469	0.142
**CEA**		2.09 ± 1.59	1.87 ± 1.53	2.00 ± 1.57	1.738	0.083
**CA153**		11.19 ± 6.11	11.39 ± 7.27	11.27 ± 6.59	-0.376	0.707
**Tumor size**		21.23 ± 8.14	20.65 ± 7.63	21.02 ± 7.96	0.946	0.344
**Distance from nipple**		2.88 ± 1.88	2.77 ± 1.89	2.84 ± 1.89	0.736	0.462
**Number of negative SLNs**		3.05 ± 1.72	3.26 ± 1.85	3.13 ± 1.77	-1.483	0.139
**Surgery**	Mastectomy	413 (63.3)	239 (36.7)	652	1.378	0.241
	Breast- conserving surgery	54 (70.1)	23 (29.9)	77		
**NSLN metastasis**	No	367 (64.5)	202 (35.5)	569	0.217	0.642
	Yes	100 (62.5)	60 (37.5)	160		
**Molecular subtype**	Luminal A	166 (63.6)	95 (36.4)	261	1.098	0.777
	Luminal B	236 (64.8)	128 (35.2)	364		
	HER2 overexpression	24 (57.1)	18 (42.9)	42		
	Triple negative	39 (66.1)	20 (33.9)	59		
	Unknown	2 (66.7)	1 (33.3)	3		
**ER**	Negative	65 (61.3)	41 (38.7)	106	0.404	0.525
	Positive	402 (64.5)	221 (35.5)	623		
**Her-2**	Negative	133 (62.1)	81 (37.9)	214	6.625	0.085
	(+)	204 (69.4)	90 (30.6)	294		
	(2+)	80 (58.4)	57 (41.6)	137		
	(3+)	50 (59.5)	34 (40.5)	84		
**Ki67**	<14%	184 (63.2)	107 (36.8)	291	0.145	0.703
	≥14%	283 (64.6)	155 (35.4)	438		
**PR**	Negative	91 (65.5)	48 (34.5)	139	0.148	0.701
	Positive	376 (63.7)	214 (36.3)	590		
**LVI**	Absent	328 (65.1)	176 (34.9)	504	0.736	0.391
	Present	139 (61.8)	86 (38.2)	225		
**Multicentric, multifocal**	No	441 (64.1)	247 (35.9)	688	0.008	0.929
	Yes	26 (63.4)	15 (36.6)	41		
**Location of primary tumor**	Other quadrant	245 (63.5)	141 (36.5)	386	0.124	0.725
	Upper outer quadrant	222 (64.7)	121 (35.3)	343		
**Histologic type**	Invasive ductal carcinoma	443 (64.8)	241 (35.2)	684	2.645	0.266
	Invasive lobular carcinoma	12 (57.1)	9 (42.9)	21		
	Other	12 (50.0)	12 (50.0)	24		

CEA, carcinoembryonic antigen; CA153, carbohydrate antigen 153; ER, estrogen receptor; HER-2, human epidermal receptor-2; LVI, lymph-vascular invasion; NSLN, non-sentinel lymph node; PR, progesterone receptor; SLNs, sentinel lymph nodes.

### Univariate and multivariate analyses for predictors of non-sentinel lymph node metastasis

**Figure 2:**
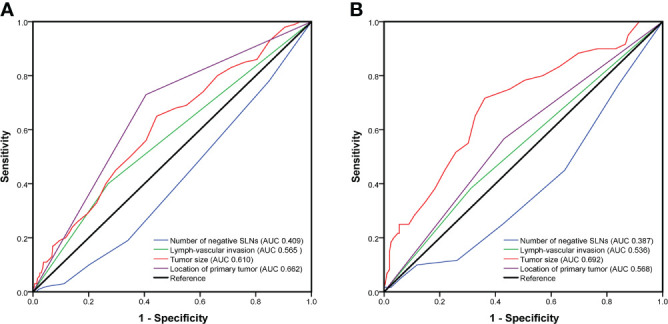
The predictive performance of four independent predictors on non-sentinel lymph node (NSLN) metastasis verified by the receiver operating characteristic (ROC) curve in the training cohort **(A)** and the validation cohort **(B)**. AUC, area under the curve; SLNs, sentinel lymph nodes.

The univariate analyses based on clinicopathological information were used to detect potential predictors of NSLN metastasis. As shown in **Table 2**, in the training cohort, only negSLN (*p*=0.001), the location of the primary tumor (*p*<0.001), LVI (*p*=0.012), and tumor size (*p*<0.001) were significantly associated with NSLN metastasis. The above variables were further incorporated into multivariate logistic regression analyses. As a result, negSLN (*p*<0.001; OR: 0.717; 95% CI: 0.604–0.851), tumor size (*p*<0.001; OR: 1.060; 95% CI: 1.030–1.091), LVI (*p*=0.028; OR: 1.757; 95% CI: 1.064–2.901), and location of the primary tumor (*p*<0.001; OR: 4.678; 95% CI: 2.784–7.862) were independent statistically significant predictors of NSLN metastasis (**Table 3**). To further verify the predictive performance of the above four independent predictors on NSLN metastasis, the ROC curve was utilized. As shown in **Figures 2A, B**, the AUC of negSLN, LVI, tumor size, and location of the primary tumor in the training cohort were 0.409, 0.565, 0.610, and 0.662, respectively. In addition, the AUC of above four independent predictors in the validation cohort were 0.387, 0.536, 0.692, and 0.568, respectively. Those results further confirmed the predictive capacity of those independent predictors on NSLN metastasis.

**Table 2:** Univariate analysis of non-sentinel lymph node (NSLN) metastasis based on clinicopathological data in the training cohort.

Clinicopathological characteristics		Non-NSLN metastasis (n=367)	NSLN metastasis (n=100)	*χ^2^/t*	p-value
**Age (years)**		53.56 ± 10.21	54.89 ± 9.35	-1.177	0.240
**CEA**		2.08 ± 1.62	2.14 ± 1.50	-0.308	0.758
**CA153**		11.02 ± 5.77	11.84 ± 7.28	-1.075	0.283
**Tumor size**		20.52 ± 7.82	23.84 ± 8.81	-3.661	0.000
**Distance from nipple**		2.83 ± 1.89	3.09 ± 1.86	-1.112	0.267
**Number of negative SLNs**		3.18 ± 1.78	2.60 ± 1.39	3.461	0.001
**Surgery**	Mastectomy	326 (78.9)	87 (21.1)	0.257	0.612
	Breast-conserving surgery	41 (75.9)	13 (24.1)		
**Molecular subtype**	Luminal A	135 (81.3)	31 (18.7)	4.844	0.184
	Luminal B	183 (77.5)	53 (22.5)		
	HER2 overexpression	15 (62.5)	9 (37.5)		
	Triple negative	32 (82.1)	7 (17.9)		
	Unknown	2 (100.0)	0 (0.0)		
**ER**	Negative	49 (75.4)	16 (24.6)	0.460	0.498
	Positive	318 (79.1)	84 (20.9)		
**Her-2**	Negative	111 (83.5)	22 (16.5)	6.653	0.084
	(1+)	161 (78.9)	43 (21.1)		
	(2+)	62 (77.5)	18 (22.5)		
	(3+)	33 (66.0)	17 (34.0)		
**Ki67**	<14%	148 (80.4)	36 (19.6)	0.616	0.432
	≥14%	219 (77.4)	64 (22.6)		
**PR**	Negative	70 (76.9)	21 (23.1)	0.186	0.666
	Positive	297 (79.0)	79 (21.0)		
**LVI**	Absent	268 (81.7)	60 (18.3)	6.377	0.012
	Present	99 (71.2)	40 (28.8)		
**Multicentric, multifocal**	No	350 (79.4)	91 (20.6)	2.852	0.091
	Yes	17 (65.4)	9 (34.6)		
**Location of primary tumor**	Other quadrant	218 (89.0)	27 (11.0)	33.080	0.000
	Upper outer quadrant	149 (67.1)	73 (32.9)		
**Histologic type**	Invasive ductal carcinoma	345 (77.9)	98 (22.1)	3.563	0.168
	Invasive lobular carcinoma	12 (100.0)	0 (0.0)		
	Other	10 (83.3)	2 (16.7)		

CEA, carcinoembryonic antigen; CA153, carbohydrate antigen 153; ER, estrogen receptor; HER-2, human epidermal receptor-2; LVI, lymph-vascular invasion; NSLN, non-sentinel lymph node; PR, progesterone receptor; SLNs, sentinel lymph nodes.

**Table 3:** Multivariate logistic regression analysis of NSLN metastasis based on clinicopathological data in the training cohort.

Clinicopathological characteristics		OR	95%CI	*P*-Value
**Number of negative SLNs**		0.717	0.604–0.851	<0.001
**Tumor size**		1.060	1.030–1.091	<0.001
**Location of primary tumor**				
	Other quadrant	1	Reference	
	Outer upper quadrant	4.678	2.784–7.862	<0.001
**LVI**				
	No	1	Reference	
	Yes	1.757	1.064–2.901	0.028

CI, confidence interval; LVI, lymph-vascular invasion; NSLN, non-sentinel lymph node; PR, progesterone receptor; SLNs, sentinel lymph nodes.

**Figure 3:**
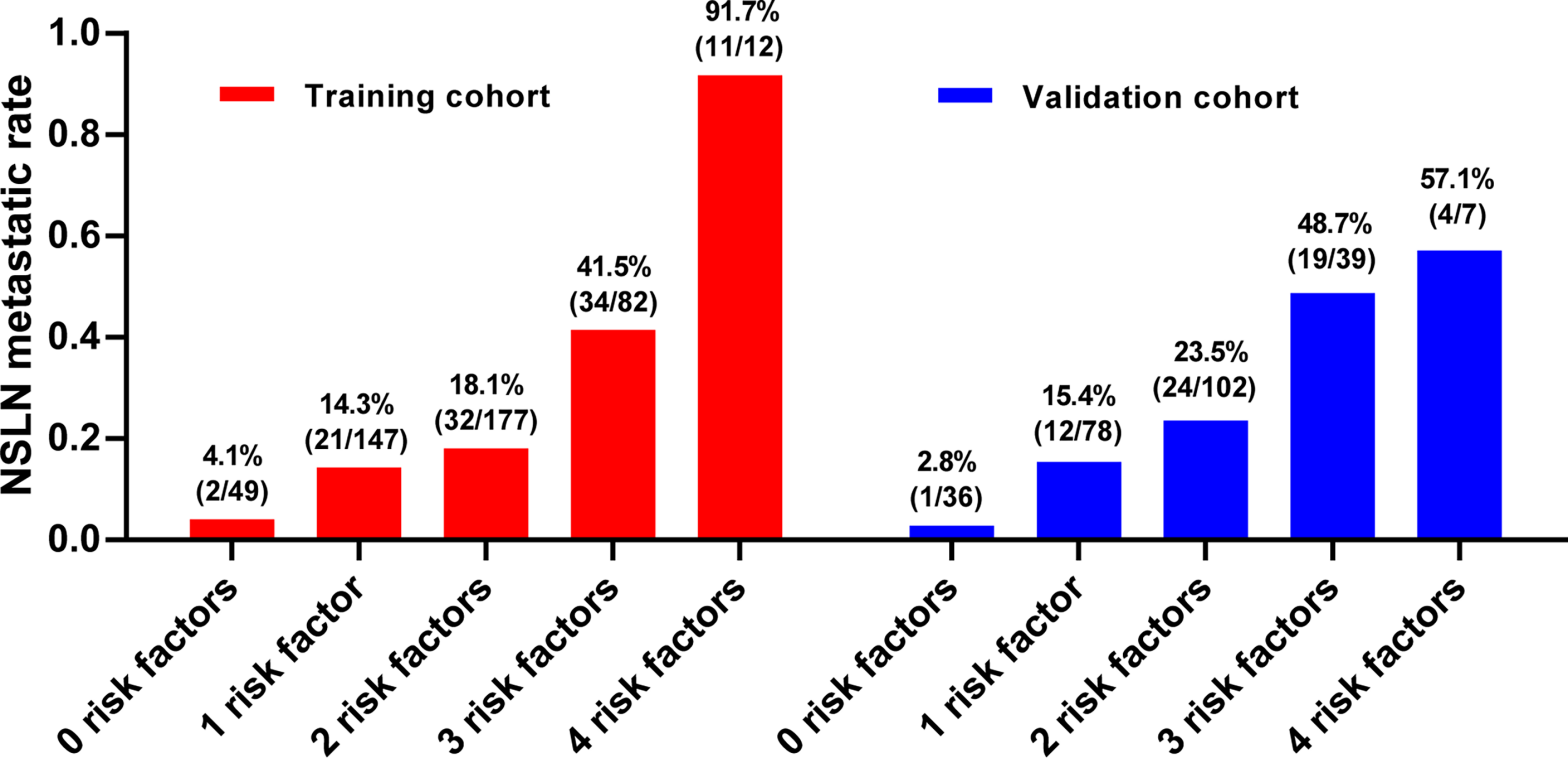
The NSLN metastatic rate when there are zero to four risk factors. The risk factors include (1): negSLN <3 (2); tumor size >20 mm (3); tumor location in the upper outer quadrant (4); presence of lymph-vascular invasion. LVI, lymph-vascular invasion; negSLN, number of negative SLNs; NSLN, non-sentinel lymph node.

The median of continuous variables’ tumor size and negSLN of the four independent predictors were calculated in the two cohorts, respectively. The median tumor size in the two cohorts was 20 mm, and the median of negSLN in the two cohorts was 3. Therefore, negSLN <3, tumor size >20 mm, tumor location in the upper outer quadrant, and presence of LVI were considered as risk factors for NSLN metastasis. To explore the NSLN metastasis of patients when multiple risk factors are superimposed, the NSLN metastatic rate was calculated in both cohorts, respectively, when zero, one, two, three, or four associated risk factors existed at the same time in one patient (**Figure 3**). In patients with none of these independent predictors, the NSLN metastatic rate was only 4.1% and 2.8% in the training and validation cohorts, respectively; when only one risk factor existed, the NSLN metastatic rate was 14.3% and 15.4%, respectively; and when two risk factors existed, these data were 18.1% and 23.5%, respectively. Meanwhile, the probabilities of NSLN metastasis were relatively high when a patient had three or all four of these risk factors, which were 41.5% and 91.7% in the training cohort and 48.7% and 57.1% in the validation cohort, respectively.

### Construction and validation of nomogram-based non-sentinel lymph node metastasis prediction

**Figure 4:**
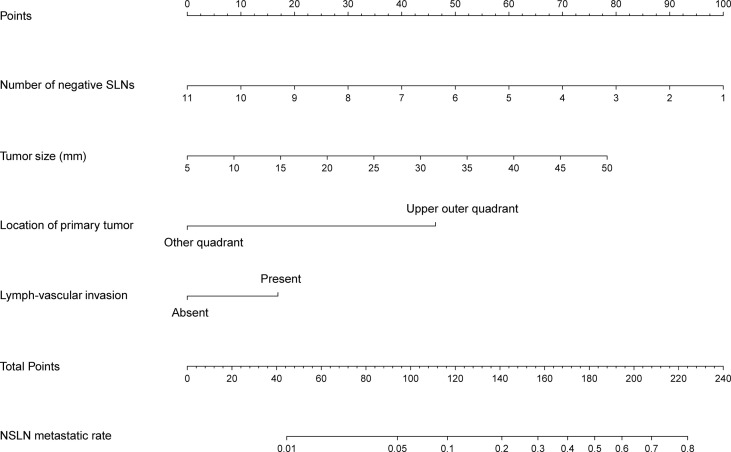
Nomogram to preoperatively estimate the risk of NSLN metastasis in T1–2 breast cancer patients with only one SLN metastasis. SLNs, sentinel lymph nodes.

**Figure 5:**
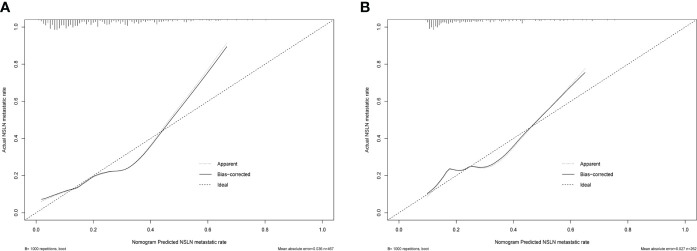
The predictive performance of the nomogram in estimating the risk of NSLN metastasis verified by the calibration curve in the training cohort **(A)** and validation cohort **(B)**. NSLN, non-sentinel lymph node.

Based on the independent predictors obtained from the multivariate analysis in the training cohort, a nomogram was established to predict the NSLN metastasis risk in T1–2 breast patients with only one SLN metastasis. As shown in **Figure 4**, negSLN exerted the largest effect on the NSLN metastasis, with a maximal score of 100 points. The effect of the tumor size also cannot be ignored, with a maximum score of 80. Furthermore, the points were approximately 45 and 20 for primary tumors located in the upper outer quadrant and with LVI, respectively. The nomogram showed good accuracy in NSLN metastasis prediction, with a C-index of 0.740. The calibration curve showed a satisfactory fit between the predictive and actual observations (**Figure 5A**).

In the validation cohort, the C-index was 0.689 for NSLN metastasis prediction based on nomogram analysis. In addition, the calibration curve also showed a good agreement of the observed NSLN metastasis risk with the predicted NSLN metastasis risk (**Figure 5B**).

### Assessment of the performance of nomogram

**Figure 6:**
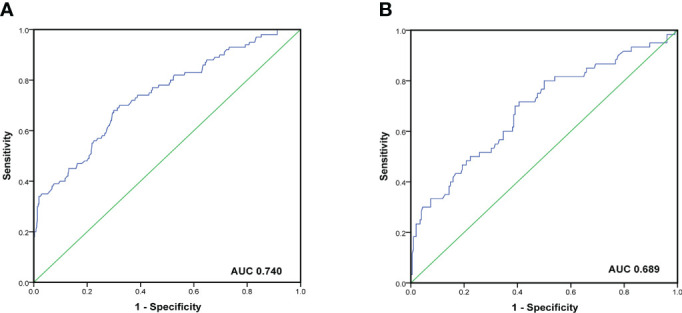
ROC curve and the area under the curves of ROC curves in the training cohort **(A)** and validation cohort **(B)**.

**Figure 7:**
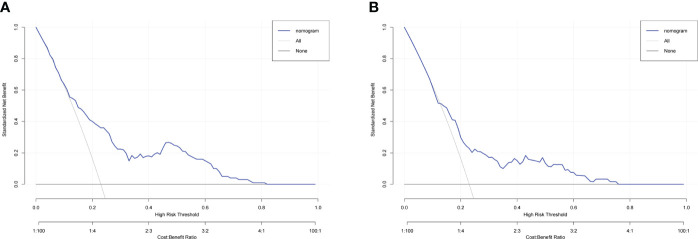
Decision curve analysis for the nomogram in predicting NSLN metastasis in the training cohort **(A)** and validation cohort **(B)**.

To evaluate the predictive capacity of the nomogram-based prediction model for the NSLN metastasis risk, the ROC curve was utilized. As shown in **Figures 6A, B**, the AUC was 0.740 (95%CI:0.683–0.798, p < 0.001) and 0.689 (95%CI: 0.608–0.770, p < 0.001) in the training and validation cohorts, respectively, indicating that the prediction model had a good predictive and discriminatory capability on NSLN metastasis. To assess the clinical validity of the nomogram, DCA was performed. As shown in the result in **Figures 7A, B**, the nomogram provided a higher standardized net benefit compared to “treat-all” and “treat-none” strategies when the risk threshold ranged approximately from 0.2 to 0.7 in both the training and validation cohorts.

## Discussion

In nearly 60% of breast cancer patients with SLN metastases, further NSLN metastasis does not exist, suggesting that routine ALND therapy in such patients is not a wise choice. In recent years, many nomograms have been established to predict the risk of NSLN metastasis in breast cancer patients with SLN metastasis, such as nomograms from MSKCC (15), Tenon (19), Cambridge (20), Stanford (21), and Helsinki University (22). Although these prediction models provided clinical decision-making for the ALND exemption of breast cancer patients with SLN metastases, their accuracy and clinical practicability have been frequently questioned in their validation over the past few years (16–18). Theoretically, patients with stage T1–2 early breast cancer with only one SLN metastasis had a lower axillary tumor load. Of the 729 such patients included in our study, only 21.9% (160/729) had NSLN metastases, making these patients more favorable candidates for selective ALND exemption. Therefore, the predictors and prediction models of NSLN metastasis in such patients need further research. However, neither a nomogram nor clinical trial has been proposed to investigate NSLN metastasis or the selective exemption of ALND in T1–2 breast cancer patients with only one SLN metastasis. Our retrospective study included clinicopathological information from 729 patients with T1–2 breast cancer with only one SLN metastasis, randomized into a training cohort (n=467) and a validation cohort (n=262). We analyzed the independent predictors of NSLN metastasis in the training cohort and established a risk prediction model. Subsequently, the satisfactory predictive capacity of the model was verified in the training and validation cohorts by the calibration curve, ROC curve, and DCA.

Through univariate analysis and multivariate logistic regression analysis in the training cohort, we identified negSLN, LVI, tumor size, and the location of the primary tumor as independent predictors of NSLN metastasis. Subsequently, the ROC curve confirmed the independent predictive capacity of these factors on NSLN metastasis in the training and validation cohorts, respectively (**Figures 2A, B**). In addition, as shown in the histogram in **Figure 3**, the more risk factors a patient has at the same time, the higher the NSLN metastatic rate will be, confirming the superposition of the NSLN metastasis risk caused by the four risk factors and providing a key indicator for clinicians to predict and judge.

The nomogram has been recognized as a user-friendly prediction tool with high accuracy and good discriminative power (35), and has been widely used to predict the prognosis and recurrence of tumor patients (36). Therefore, based on these independent predictors, we established a nomogram to predict the risk of NSLN metastasis in T1–2 breast cancer patients with only one SLN metastasis. The C-index and AUC of the prediction model were 0.740, which showed a reasonable discrimination capacity. The consistency between the predicted value and the observed value of the calibration curve also confirms the predictive capacity of the prediction model. Subsequently, we externally validated the model with the clinicopathological information of patients in the validation cohort. The results show that the C-index and AUC of the prediction model in the validation are 0.689; the calibration curve also shows the consistency between the predicted value and the observed value. These show that the prediction model also has satisfactory discrimination and prediction capacity in the verification cohort.

Compared with the MSKCC nomogram containing eight predictors, our nomogram has achieved satisfactory NSLN metastasis prediction on the premise of incorporating four variables, which enhances the practicability of our model. Among these four predictors, the tumor size, negSLN, and LVI have been included in multiple NSLN metastasis prediction models in previous studies (15, 21, 22, 37).

The tumor size has long been the main predictor of NSLN metastasis in several previous studies (37–39). In the nomogram from Tenon Hospital, the risk of NSLN metastasis in breast cancer patients with a tumor size between 11 and 20 mm and a tumor size >20 mm was 1.5 and 3 times higher than that in patients with a tumor size ≤10 mm (p = 0.006), respectively (19). The nomogram from Helsinki University Central Hospital also identified the tumor size as an independent predictor of NSLN metastasis (OR = 1.021; 95% CI 1.008–1.034; p=0.003), meaning that the risk of NSLN metastasis is 1.021 times higher for each 1-mm increase in tumor size. Consistently, tumor size as a continuous variable was also considered to be an independent predictor of NSLN metastasis in our study (OR = 1.060; 95% CI 1.030–1.091; *p*<0.001), meaning that for every 1-mm increase in the tumor size, the risk became 1.060 times higher than before. To investigate the NSLN metastatic rate in patients with different tumor size ranges, we further subdivided all patients into five subgroups for every 10 mm according to the tumor size and analyzed the relationship between the NSLN metastatic rate and the tumor size in the whole cohort (**Table 4**). The results showed a significant positive correlation between the tumor size and the NSLN metastatic rate. In addition, it is worth noting that the NSLN metastatic rate in patients with a tumor size ≤10 mm was only 2.6%, which showed that such patients could be completely exempted from ALND to a certain extent.

**Table 4:** Relationship between tumor size and NSLN metastasis in the whole cohort.

Tumor size (mm)	NSLN metastatic rate
0–10	2.6% (1/39)
11–20	14.7% (51/346)
21–30	28.6% (73/255)
31–40	38.0% (27/71)
41–50	44.4% (8/18)

NSLN, non-sentinel lymph node.

The more positive SLNs, the greater the risk of NSLN metastasis, which has been confirmed in previous studies (22, 37, 40). The SLN metastatic rate has also been identified to be an independent predictor of NSLN metastasis (19, 20, 41). In our study, the number of metastatic SLNs was locked as 1, which resulted in only negSLN being included as a variable for analysis. Multivariate regression analysis showed that negSLN is an independent predictor of NSLN metastasis (OR = 0.717; 95% CI 0.604-0.851; *p*<0.001). Then, the nomogram indicates that negSLN was negatively correlated with the risk of NSLN metastasis in the case of one SLN metastasis, which showed the same trend as previous studies.

Previous studies have shown that breast cancer patients with the primary tumor in the outer upper quadrant have a higher rate of recurrence and metastases (42, 43). However, whether the primary tumor location can influence the NSLN status has long been controversial. In the earlier proposed nomograms, such as MSKCC, Tenon, and Cambridge, the location of the primary tumor was not included in the analysis, which may be caused by the incomplete clinicopathological information of the patient. With the development of the nomograms of NSLN prediction, the tumor location has been gradually included in the analysis and discussion but has not yet been considered as an independent predictor of NSLN metastasis (32, 37). In our study, multivariate logistic regression analysis showed that the location of the primary tumor was an independent predictor of NSLN metastasis (OR = 4.678; 95% CI 2.784-7.862; *p*<0.001), indicating that the NSLN metastasis risk of the primary tumor in the upper outer quadrant was 4.678 times higher than the other quadrants of the primary tumor. As shown in **Figure 2**, in the training and verification cohorts, the ROC curve confirmed the independent discrimination and predictive capacity of the location of the primary tumor for NSLN metastasis, with AUC values of 0.662 and 0.568, respectively.

Tumor cells invade the lymphatic vessels and drain to the axillary region, leading to axillary lymph node metastasis in breast cancer. The LVI was an independent predictor of NSLN metastasis in MSKCC, Stanford, and Helsinki models (15, 21, 22), which was also confirmed in our study (OR = 1.757; 95% CI 1.064–2.901; *p*<0.028). In the MSKCC and Stanford models, 40.5% and 44.6% of the patients were found to have NSLN metastasis, respectively (15, 21). In our study, the NSLN metastatic rate was only 29.8% and 32.8% in the training and validation cohorts, respectively. This may be because the patients included in our study are at an earlier stage (T1–2 and only one SLN metastasis) than MSKCC and Stanford models (the number of SLN metastasis is not limited).

In addition, in previous studies, the histologic type, hormonal receptor, and HER-2 status were identified to be associated with NSLN metastasis (44, 45). Unfortunately, none of these have been confirmed in current studies. Whether the molecular-subtype classifications of breast cancer can be an independent predictor of NSLN metastasis remains a controversial topic. Some previous studies have supported the molecular subtype as a predictor of NSLN metastasis and incorporated it into the nomogram, and satisfactory prediction results were obtained (46, 47). However, many prediction models for NSLN metastasis, including MSKCC, Tenon, Cambridge, and Helsinki models, do not take the molecular subtype as one of the criteria (15, 19, 22). In our study, the molecular subtype was also not an independent predictor of NSLN metastasis (*p*=0.168), so we did not incorporate it in our prediction model.

To further assess the clinical application value of this nomogram-based model, DCA was used to calculate the standardized net benefit, where the risk threshold between 0.2 and 0.7 indicated a greater net benefit. In addition, previous studies have shown that in patients with SLN metastases, most surgeons will exempt further ALND if the predicted probability of NSLN metastasis is 10% or less (48). When the predicted cutoff value in our model was 10%, the false-negative rate, sensitivity, and specificity of the prediction model were 13.0%, 87.0%, and 45.4% in the training cohort, respectively. The optimal cutoff value of the model obtained by the Youden index was 23%. Additionally, in this case, the false-negative rate, sensitivity, and specificity of the prediction model were 32.0%, 68.0%, and 70.0% in the training cohort, respectively. Considering the clinical application, false-negative rate, specificity, and sensitivity, the cutoff value of the model is finally set to 10%, which means that if the NSLN metastatic rate was less than 10% according to our prediction model, the exemption of ALND would be recommended. Therefore, based on the above analysis, we believe that our nomogram can serve as a preoperative predicting method to assess the NSLN metastasis risk in T1–2 breast cancer patients with only one SLN metastasis and provide important guidance and advice for clinicians’ decision-making.

However, there are still some limitations in our study. Firstly, this study is retrospective and cannot support the establishment of causality. Secondly, due to incomplete sample information, it was not further identified whether the SLN metastasis size and histological grade were related to NSLN metastasis, although previous studies did not support their correlation. Thirdly, NSLNs are mainly examined by routine histopathology, which may underestimate NSLN metastasis. Finally, this is a single-center retrospective study; future multicenter prospective studies in a larger patient cohort are needed to validate our prediction model.

## Conclusion

In conclusion, this study is the first to reveal the independent predictors of NSLN metastasis in T1–2 breast cancer patients with only one SLN metastasis, which were negSLN, location of the primary tumor, LVI, and tumor size. Subsequently, based on these independent predictors, a risk prediction model for NSLN metastasis was established and verified in the training and validation cohorts. C-index, ROC curve, calibration curve, and clinical decision curve confirmed the satisfactory predictive capacity and clinical application capacity of the model. We believe that this nomogram-based prediction model could provide an optimal preoperative estimation of the NSLN metastasis risk in T1–2 breast cancer patients with only one SLN metastasis, thereby facilitating precise clinical decision-making on the selective exemption of ALND in such patients. However, the model requires further validation by multicenter prospective studies in a larger patient cohort.

## Data availability statement

The raw data supporting the conclusions of this article will be made available by the authors, without undue reservation.

## Ethics statement

This study was reviewed and approved by The Ethics Committee of Harbin Medical University Cancer Hospital. The patients/participants provided their written informed consent to participate in this study.

## Author contributions

SX and YY provided direction and guidance throughout the preparation of this manuscript. LL analyzed and interpreted the patient, tumor, and risk factor data as well as drafted the manuscript. GL and YL generated the figures and made significant revisions to the manuscript. LZ, XZ, JW, and XW provided patient data and clinical information. All authors contributed to the article and approved the submitted version.

## Funding

This work was funded by the National Natural Science Foundation of China (grant numbers 82072904, 81872149), the Outstanding Youth Project of Heilongjiang Provincial Natural Science Foundation (grant number YQ2019H027), the Distinguished Young Scholars of Harbin Medical University Cancer Hospital (grant number JCQN2018-03), the Young Elite Training Foundation Grant of Harbin Medical University Cancer Hospital (grant number JY2016-02), and the Postgraduate Practice Innovation Project of Harbin Medical University (grant number YJSCX2020-120HYD).

## Conflict of interest

The authors declare that the research was conducted in the absence of any commercial or financial relationships that could be construed as a potential conflict of interest.

## Publisher’s note

All claims expressed in this article are solely those of the authors and do not necessarily represent those of their affiliated organizations, or those of the publisher, the editors and the reviewers. Any product that may be evaluated in this article, or claim that may be made by its manufacturer, is not guaranteed or endorsed by the publisher.
